# Sodium butyrate induces mitochondrial pathway apoptosis in liver cancer via ATF4/SLC7A11-mediated ferroptosis

**DOI:** 10.1371/journal.pone.0353653

**Published:** 2026-07-15

**Authors:** Xiaolan Meng, Yubin Li, Miao He, Chen Wang, Haoran Shengsong, Jiacheng Guo, Jiayu Zhang, Shuang Zhao, Yulei Zhu, Bowen Li, Yuqiang Li, Hongzhi Sun

**Affiliations:** 1 The First Affiliated Hospital of Jinzhou Medical University, Jinzhou Medical University, Jinzhou, Liaoning, China; 2 Clinical Biobank Center, the First Affiliated Hospital of Jinzhou Medical University, Jinzhou, Liaoning, China; 3 Key Surgical Laboratory of Educational Administration of Liaoning Province, Jinzhou, Liaoning, China; 4 Key Laboratory of Liaoning Tumor Clinical Metabolomics (KLLTCM), Jinzhou, Liaoning, China; Fudan University, CHINA

## Abstract

Liver cancer, a prevalent and aggressive malignancy globally, is associated with high morbidity and mortality rates. Butyrate, a metabolite produced by intestinal microbiota, is capable of restricting cancer initiation and progression. However, the precise mechanisms underlying its effects on liver cancer remain poorly understood. This study utilized a CCK-8 cytotoxicity assay to demonstrate that sodium butyrate (NaB) suppresses liver cancer cell proliferation through ferroptosis and apoptosis. The involvement of ATF4/SLC7A11 signaling and mitochondrial dysfunction in NaB-induced ferroptosis and apoptosis was further investigated. Results revealed a decrease in ATF4 and SLC7A11 expression, an elevation in the levels of malondialdehyde (MDA) and reactive oxygen species (ROS), and a reduction in glutathione (GSH) in NaB-treated liver cancer cells. These ferroptosis-related alterations could be reversed by an ATF4 activator. Additionally, NaB-treated liver cancer cells presented a decrease in mitochondrial membrane potential (MMP), accumulation of mitochondrial ROS, and mitochondrial damage. These cellular changes disrupted the BAX/BCL-2 balance, leading to cytochrome C release, which subsequently activated caspase9 and caspase3, initiating mitochondrial pathway apoptosis. *In vivo*, NaB treatment resulted in increased iron content in liver cancer tissues, along with upregulated cytochrome C, activated caspase9, and caspase3 expression; these effects were counteracted by ferrostatin-1 (Fer-1). Collectively, this study elucidates that NaB induces mitochondrial damage *via* ferroptosis mediated by ATF4/SLC7A11, ultimately triggering mitochondrial pathway apoptosis in hepatoma cells. These findings may offer novel insights into therapeutic strategies for hepatoma.

## Introduction

The most recent data indicate that liver cancer is the 3rd leading cause of cancer-related mortality around the world, while in China, it has become the 2nd leading cause of cancer death [1]. Among these, hepatocellular carcinoma (HCC) represents 80% of all primary liver cancers [2]. Besides hepatitis B virus (HBV) and hepatitis C virus (HCV), factors such as excessive alcohol consumption, metabolic syndrome, type 2 diabetes, obesity, aflatoxin exposure, and nonalcoholic fatty liver disease are also significant risk factors for liver cancer [3]. These risk factors, closely intertwined with lifestyle choices, contribute to liver cancer development by disrupting the balance between the activation and inactivation of oncogenes and tumor suppressor genes [4]. Although surgical resection, radiotherapy, and chemotherapy have made certain advances, the five-year survival rate for patients suffering liver cancer and intrahepatic cholangiocarcinoma remains a dismal 20.8% as of August 2022 [5]. Consequently, it is paramountly important to develop safer therapeutic agents with stronger effectiveness.

Short-chain fatty acids (SCFAs, butyrate, propionate, and acetate, etc.) are metabolites from gut microbiota that have been shown to protect normal tissues and inhibit cancer progression [6–8]. These compounds can restrict histone deacetylase activity and promote G protein-coupled receptors (GPCRs) to be activated [9]. Among SCFAs, butyrate is a potent tumor suppressor in colorectal cancer [10–12], gastric cancer [13], cervical cancer [14], lung cancer [15] and other cancer types. Nevertheless, its therapeutic effects and underlying mechanisms in liver cancer remain underexplored.

Ferroptosis and apoptosis are distinct forms of regulated cell death. Ferroptosis is an iron-dependent cell death mechanism identified recently, and features excessively accumulated lipid peroxides and reactive oxygen species (ROS) [16]. Apoptosis, a key component of numerous cellular processes, involves the endogenous mitochondrial pathway [17]. Upon oxidative stress, such as ROS accumulation, the pro-apoptotic protein BAX is activated, leading to increased mitochondrial outer membrane permeability. Simultaneously, downregulation of BCL-2, an anti-apoptotic protein, fails to inhibit BAX activity, as a result, cytochrome C can be released from the mitochondria into the cytoplasm, which activates initiator and effector caspases, ultimately driving mitochondrial pathway apoptosis [18,19].

According to previous studies, ATF4/SLC7A11 could critically prevent hepatocarcinogenesis [20], however, whether the ATF4/SLC7A11 axis participates in the inhibition of hepatoma cell proliferation by butyrate remains unclear. Therefore, this study investigates how sodium butyrate (NaB) inhibits liver cancer cell proliferation, elucidates the function of the ATF4/SLC7A11 pathway in NaB-induced ferroptosis, and explores the function and mechanisms of mitochondrial involvement in NaB-induced ferroptosis and apoptosis. The findings aim to assist researchers in developing valuable therapeutic strategies targeting liver cancer from new perspectives.

## Methods

### Cell culture

Human HCC cell lines HepG2 (KALANG, KL-C1172H, China) and Huh7 (Pricella, CL-0120, China) were cultured in DMEM medium (Pricella, PM150210, China) supplemented with 10% FBS (Clark, FB25015, USA) and 1% penicillin/streptomycin solution (Beyotime, C0222, China) in a cell incubator (Thermo, HERACELL150i/240i, USA) at 37°C with 5% CO₂. The cells were cultured to the logarithmic growth phase for subsequent experiments.

### Cell viability assay

Experimenters seeded HepG2 and Huh7 cells in the logarithmic growth phase into 96-well plates (6 × 10³ cells/well). Upon cell adhesion, 10 µL of CCK-8 solution (APExBIO, K1018, USA) and 100 µL of DMEM complete medium were added to each well, followed by treatment with the corresponding drugs for 24 and 48 hours. The cells underwent 2–4 h of incubation at 37°C in a cell incubator with 5% CO₂ in the dark. A microplate reader (Bio-Rad, iMark, USA) was employed for the measurement of the absorbance at 450 nm.

### Colony formation assay

Experimenters seeded HepG2 and Huh7 cells into six-well plates (1 × 10³ cells/well). After adherence, the cells received 48 h of treatment using corresponding drugs and two times of PBS washes (Pricella, PB180327, China), followed by being added with a fresh complete medium. The cells underwent one week of culture at 37°C. Then, the cells received 10–20 minutes of fixation treatment in 1 mL of 4% paraformaldehyde (PFA, Beyotime, P0099, China) at room temperature (RT), followed by 10–20 minutes of staining in 1% crystal violet solution (Beyotime, C0121, China) in the dark. After the removal of the solution and ddH₂O wash, the wells were inverted to dry, and photographed for analysis.

### Bioinformatics analysis

The dataset (GSE213944) from the GEO database was filtered, focusing on the data from the first three days. The FerrDb database (http://www.zhounan.org/ferrdb/legacy/index.html) was adopted to identify ferroptosis-related genes. Heat maps, volcano plots, and Gene Ontology (GO) biological process (BP) pathway enrichment analyses relied on RStudio software (R4.2.1). STRING (https://string-db.org) served for analyzing the protein-protein interaction (PPI) network of ferroptosis-associated genes and Cytoscape software (Cytoscape_v3.10.2) served for corresponding visualization. Two key central protein interaction networks were identified using MCODE, comprising 8 ferroptosis marker genes, 8 driver genes, and 12 suppressor genes (Table 1), the classification of genes refers to the FerrDb database (http://www.zhounan.org/ferrdb/legacy/index.html). HSPB1/HMOX1 and ATF4/SLC7A11 were selected as key ferroptosis marker genes with roles in promoting or inhibiting ferroptosis. Survival analysis data for ATF4 were obtained from clinical liver cancer samples in the TCGA database and visualized using RStudio software. A scatter plot showing the correlation between ATF4 and SLC7A11 was drawn using the GEPIA2 website (http://gepia2.cancer-pku.cn/#index).

**Table 1:** Genes of two key central protein interaction networks.

Marker	Driver	Suppressor
HSPB1	HMOX1	HSPB1
HMOX1	EGFR	ATF4
ATF4	PIK3CA	SLC7A11
SLC7A11	TP53	MUC1
ALB	CDKN2A	SRC
AURKA	G6PD	TP53
SRXN1	ATM	SQSTM1
PRDX1	KRAS	CAV1
		JUN
		GCLC
		STAT3
		CDKN1A

### Western blotting

HepG2 and Huh7 cells underwent lysis treatment on ice with prepared RIPA buffer (Beyotime, P0013, China). Centrifugation at 4°C was followed by the collection of the supernatant. The BCA assay kit (Beyotime, P0010, China) assisted in determining the total concentration. The molecular weight of the target proteins was taken into account to perform electrophoresis with SDS-PAGE gels of varying concentrations. Proteins were moved to PVDF membranes which received 15 min of blockage in rapid blocking solution, TBST wash, one night of incubation using primary antibodies at 4°C, and another three times of TBST washes. Membranes were then subjected to 2 h of incubation with secondary antibodies at RT, followed by another three times of TBST washes. Visualization of protein bands relied on the ECL chemiluminescence. Ferroptosis-related proteins (ATF4, Boster, BM5179; SLC7A11, Proteintech, 26864–1-AP; GPX4, Proteintech, 30388–1-AP) and apoptosis-related proteins (Bax, Bcl-2, cytochrome C, cleaved caspase-9, Boster, BA0315−2, A00040-2, PB9334, PB0285; cleaved caspase-3, Proteintech, 19677–1-AP) were detected as described.

### MDA and GSH assays

Logarithmically growing HepG2 and Huh7 cells in 6-well plates received 48 h of treatment using varying concentrations of NaB (HepG2: 0, 5, 10 mM; HuH7: 0, 10, 20 mM) and/or the ATF4 activator E235 (10 µM). Following PBS washes, the samples were analyzed for MDA and GSH levels using respective assay kits (Nanjing Jiancheng, A003-4–1, A006-2–1, China) as per the producer’s protocol, and absorbance readings were taken at the corresponding wavelengths via a microplate reader.

### ROS detection

Experimenters diluted the DCFH-DA probe (Beyotime, S00335-1, China) in serum-free medium to a final concentration of 5 µM (1:1000). Logarithmically growing HepG2 and Huh7 cells were treated with different concentrations of NaB (HepG2: 0, 5, 10 mM; HuH7: 0, 10, 20 mM) and/or ATF4 activator E235 (10 μM) for 48 hours. After removing the culture medium, each well was added with 1 mM of the probe solution to receive 30 min of incubation at 37°C in the dark. The unincorporated probe was washed out with a serum-free medium, and fluorescence was measured using a fluorescence microscope (Olympus IX73, Japan).

### Annexin V-PE apoptosis assay

Logarithmically growing HepG2 and Huh7 cells were exposed to different NaB concentrations (HepG2: 0, 5, 10 mM; HuH7: 0, 10, 20 mM) and/or the ferroptosis inhibitor Fer-1 (2 µM, APExBIO, A4371, USA) for 48 hours. Following medium removal, cells underwent PBS wash, and 195 µL and 5 µL of Annexin V-PE binding solution were added to each well in succession (Beyotime, C1065L, China). After 20 min of incubating at RT in the dark, fluorescence images were captured using a fluorescence microscope.

### TUNEL apoptosis assay

TUNEL assays (Beyotime, C1086, China) were conducted as per the producer’s protocol. Logarithmically growing HepG2 and Huh7 cells underwent 48 h of treatment using various NaB concentrations (HepG2: 0, 5, 10 mM; HuH7: 0, 10, 20 mM) and/or Fer-1 (2 µM). Cells were subjected to 30 min of fixation treatment in 4% PFA at RT, HBSS wash (Beyotime, C0218, China), and 5 min of permeabilization with PBS that contained 0.3% Triton X-100 (Solarbio, T8200, China) successively. After washing twice with HBSS, each cell was added with 50 µL of the prepared TUNEL solution, covered with a circular plastic sheet for the prevention of evaporation, followed by 1 h of incubation at 37°C in the dark. Following three HBSS washes, fluorescence was observed, together with the capturing of images by virtue of a fluorescence microscope. Negative controls are achieved by omitting TdT from the reaction mixture; positive controls are obtained by pretreatment with DNase I. All experimental groups were processed in parallel with the controls.

### Transmission electron microscopy (TEM)

HepG2 and Huh7 cells were divided into three experimental groups: control, NaB, and NaB + Fer-1. Following treatment, cells underwent centrifugation and fixation treatment in an electron microscope fixative at 4°C. After agar pre-embedding, the cells were fixed with 1% osmium tetroxide (Ted Pella Inc., USA) prepared in 0.1M phosphate buffer (pH 7.4) at RT, protected from light, for 2 hours, which then received three washes in 0.1M phosphate buffer (pH 7.4), dehydration at RT, osmotic embedding, and polymerization. An ultrathin sectioning machine (Leica UC7, Germany) was used to achieve sections of 60–80 nm thickness. A TEM (Hitachi HT7800/HT7700, Japan) was used to observe the stained sections.

### Mitochondrial ROS (mtROS) detection

Logarithmically growing HepG2 and Huh7 cells were subjected to 48 h of treatment using different concentrations of NaB (HepG2: 0, 5, 10 mM; HuH7: 0, 10, 20 mM) and/or Fer-1 (2 µM). After removing the medium, the cells underwent three washes in serum-free medium before half an hour of incubation using 10 µmol/L pre-diluted MitoROS stain (Dojindo, MT148, Japan) in a cell culture incubator at 37°C, 5% CO₂, protected from light. Following two washes with HBSS, a fluorescence microscope was adopted to capture the fluorescence images.

### Mitochondrial membrane potential (MMP) detection

JC-1 working solution (Dojindo, MT09, Japan) was prepared at a final concentration of 2 µmol/L as per the producer’s protocol. Logarithmically growing HepG2 and Huh7 cells were subjected to 48 h of treatment using different concentrations of NaB (HepG2: 0, 5, 10 mM; HuH7: 0, 10, 20 mM) and/or Fer-1 (2 µM). Treated cells were washed twice with HBSS before half an hour of incubation using the prepared JC-1 working solution at 37°C in a 5% CO₂ incubator in the dark. After two washes with HBSS, Imaging Buffer Solution diluted with ultrapure water was added. A fluorescence microscope was employed for the observation of the cells, capturing red (indicative of normal MMP) and green (indicative of decreased MMP) fluorescence images.

### Animal models

*In vivo* experiments adopted 4-week-old female BALB/c nude mice (Liaoning Changsheng Biotechnology, Production License No. SCXK (Liao) 2020−0001), as female mice are generally more tolerant to tumor engraftment and less aggressive in inter-male fighting, which can affect tumor growth and welfare. Animal studies were conducted in accordance with the guidelines for the care and use of laboratory animals and were approved by the Ethics Committee of Jinzhou Medical University (Ethics Review Form No. 240135). It is confirmed that the study was reported according to ARRIVE guidelines. The mice were housed under standard conditions at the Animal Experimentation Center of Jinzhou Medical University, with a 12-h light/dark cycle and free access to food and water. After a one-week acclimatization period, each mouse was administered 5 × 10^6^ Huh7 cells by subcutaneous injection into the left axilla. With the tumors being clear and distinct, the mice fell into three groups in a random manner (n = 5 per group): Control, NaB, and NaB + Fer-1. The NaB group was administered 500 mg/kg NaB daily by gavage, the NaB + Fer-1 group received 500 mg/kg NaB daily by gavage and 10 mg/kg Fer-1 intraperitoneally every three days, and the control group received equal volumes of normal saline by gavage and intraperitoneal injection. Two weeks after the treatment, the mice were anesthetized with carbon dioxide and euthanized through cervical dislocation. The tumors were then excised and subjected to subsequent experiments. Tumor volume = length × width × width/2.

### Hematoxylin-eosin (HE) staining

The excised tumors underwent fixation treatment in 4% PFA and paraffin embedding before being sectioned. The sections were subjected to xylene dewaxing, ethanol rehydration, and three times of PBST wash (each lasting 5 minutes) in succession, followed by 3 minutes of staining using hematoxylin solution (Beyotime, C0105S, China) and 5 minutes of rinsing in running water. The sections then underwent 20 seconds of differentiation in hydrochloric acid ethanol, rinsing in running water, and 2 minutes of eosin staining. After staining, sections were dehydrated through absolute ethanol after washing with running water. Neutral gum was used to seal the sections after permeabilization with xylene, and a microscope was employed for the observation of relevant images.

### Prussian blue staining

Tissue sections were dewaxed, rehydrated, soaked, and washed in distilled water for 3 minutes. 30 minutes of Peris staining was performed using a working solution prepared from the Prussian blue kit (Solarbio, G1422, China) at RT, protected from light. After the staining solution was removed, the sections received 10 minutes of washing in distilled water and 8 minutes of staining with nuclear solid red solution successively. After 2–3 seconds of rinsing in running water, the sections were dehydrated, and permeabilized with absolute ethanol and xylene, sealed with neutral gum, air-dried at RT, and observed by virtue of a microscope.

### Immunohistochemical (IHC) staining

For IHC staining, the prepared sections, after 1 h of baking at 60°C, underwent xylene deparaffinization, and ethanol hydration in succession. After washing with PBST, the sections were heated at 95°C for 10 minutes to achieve antigen retrieval. Following cooling to RT, the sections underwent 10 minutes of incubation with 3% hydrogen peroxide solution for the blockage of endogenous peroxidase activity, followed by being washed with PBST, and circled with an immunohistochemistry pen. 45 minutes of blocking was carried out using goat serum at RT, followed by removal of the blocking solution. The sections were subjected to one night of incubation using primary antibodies against Cytochrome C (1:200), cleaved caspase-9 (1:200), and cleaved caspase-3 (1:200) in a humidified chamber at 4°C. The next day, after 30 minutes of rewarming at RT and PBST wash, the sections underwent 30 minutes of incubation using secondary antibody at RT, and then were washed again. After 3 minutes of staining with DAB working solution (Beyotime, P0203, China) at RT (protected from light), the sections received 3 minutes of counterstaining with hematoxylin. After dehydration and permeabilization treatment, the sections were sealed, and images were captured for microscopic observation.

### Statistical analysis

Statistical analysis relied on GraphPad Prism 9. The t-test and one-way ANOVA served for comparisons between two groups and among multiple groups, respectively. Data presentation followed mean ± standard deviation (SD) format. *P* < 0.05 indicated statistical significance.

## Results

### NaB inhibits the proliferation of hepatoma cells HepG2 and Huh7 by inducing ferroptosis and apoptosis

**Fig 1:**
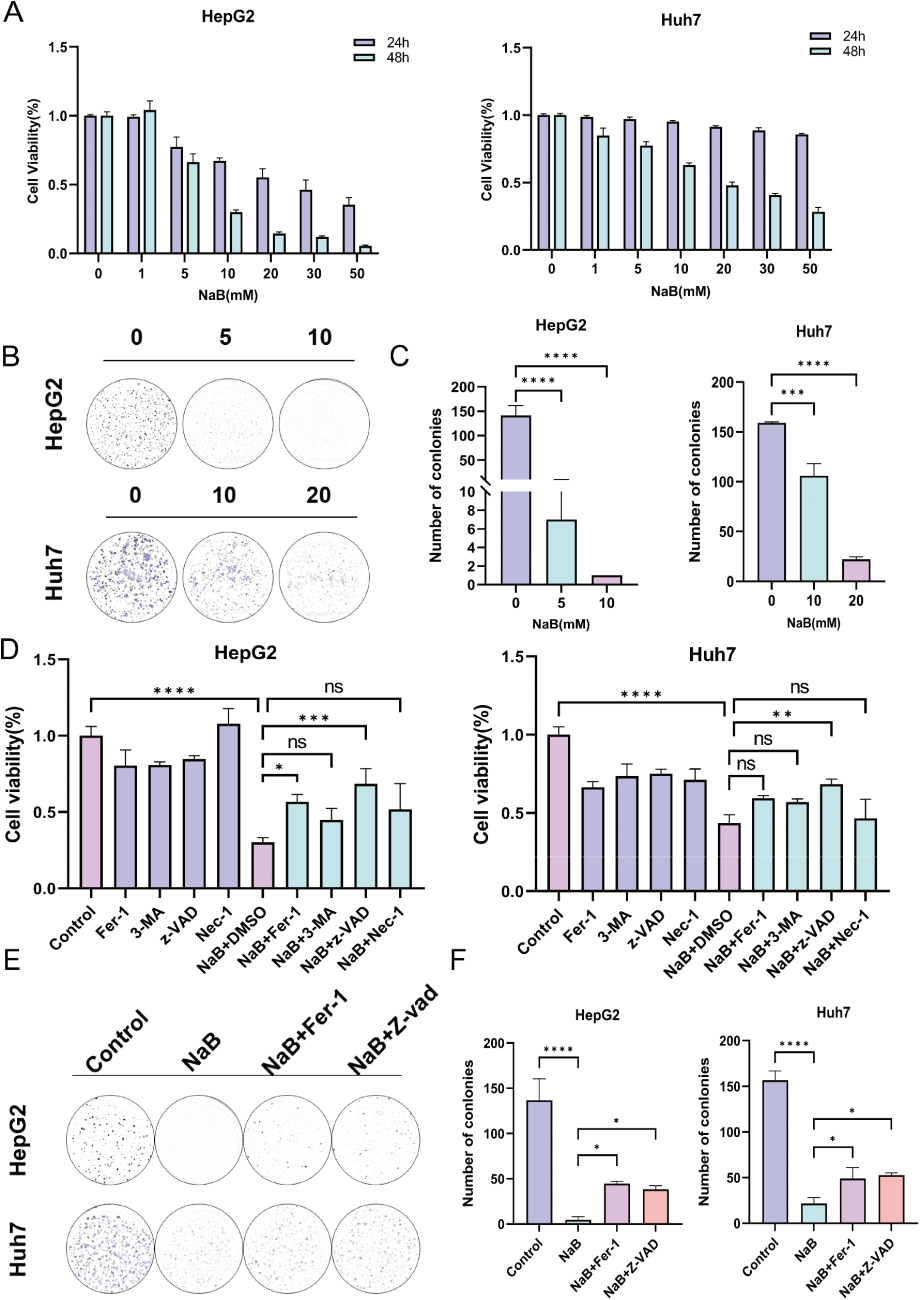
NaB inhibits hepatoma cell proliferation by inducing ferroptosis and apoptosis. (A) Cell viability of HepG2 and Huh7 cells treated with varying NaB concentrations (0, 1, 5, 10, 20, 30, 50 mM) for 24 and 48 hours was assessed via the CCK-8 assay. (B, C) NaB treatment suppressed the clonogenic ability of HepG2 and Huh7 cells after 48 hours. (D) The impact of Fer-1 (2 μM), z-VAD (30 μM), 3-MA (5 mM), Nec-1 (30 μM), and/or NaB (HepG2: 10 mM, Huh7: 20 mM) on cell viability after 48-hour treatment. (E, F) The effect of Fer-1 (2 μM), z-VAD (30 μM), and/or NaB on cell clonogenic proliferation after 48-hour treatment. The presentation of results follows mean  ±  standard deviation (SD) format, with the number of biological replicates (n) = 3, One-way ANOVA was conducted for (Panel C,D and F). *p  <  0.05, **p  <  0.01, ***p  <  0.001, ****p  <  0.0001.

CCK-8 assay data (Fig 1A) revealed that varying doses of NaB could suppress HepG2 and Huh7 cell proliferation after 24 and 48 h of treatment. After calculation, the IC50 of HepG2 cells treated with sodium butyrate for 24 hours and 48 hours was 24.63 mM and 7.055 mM, respectively. The IC50 values of Huh7 cells were 618.1 mM and 18.28 mM, respectively. The effective concentration of sodium butyrate that causes half of HepG2 cells to die is lower, indicating that HepG2 cells are more sensitive to the treatment with sodium butyrate. The colony formation assay (Fig 1B and 1C) further demonstrated that NaB significantly impaired the clonogenic ability of hepatoma cells. To investigate the mechanisms underlying NaB’s antiproliferative effects, ferrostatin-1 (Fer-1, a ferroptosis inhibitor), 3-Methyladenine (3-MA, an autophagy inhibitor), z-VAD-FMK (z-VAD, an apoptosis inhibitor), and necrostatin-1 (Nec-1, a necrosis inhibitor) were co-incubated with NaB in the CCK-8 assay. The results (Fig 1D) showed a significant reversal of NaB-induced proliferation inhibition in HepG2 cells when co-treated with Fer-1 or z-VAD, and the NaB + z-VAD group presented a similar restoration of proliferation inhibition in Huh7 cells to that in the NaB-only group. Additionally, colony formation assays (Fig 1E and 1F) confirmed that the NaB group presented markedly reduced clonogenic potential of hepatoma cells versus the control group. However, there were more clones in the NaB + Fer-1 and NaB + z-VAD groups relative to the NaB-only group, further suggesting that ferroptosis and apoptosis contribute significantly to NaB-mediated suppression of cell proliferation. Overall, these results indicate that NaB inhibits hepatoma cell proliferation through the induction of ferroptosis and apoptosis.

### Bioinformatics screening for differentially expressed genes (DEGs) of ferroptosis before and after NaB treatment

**Fig 2:**
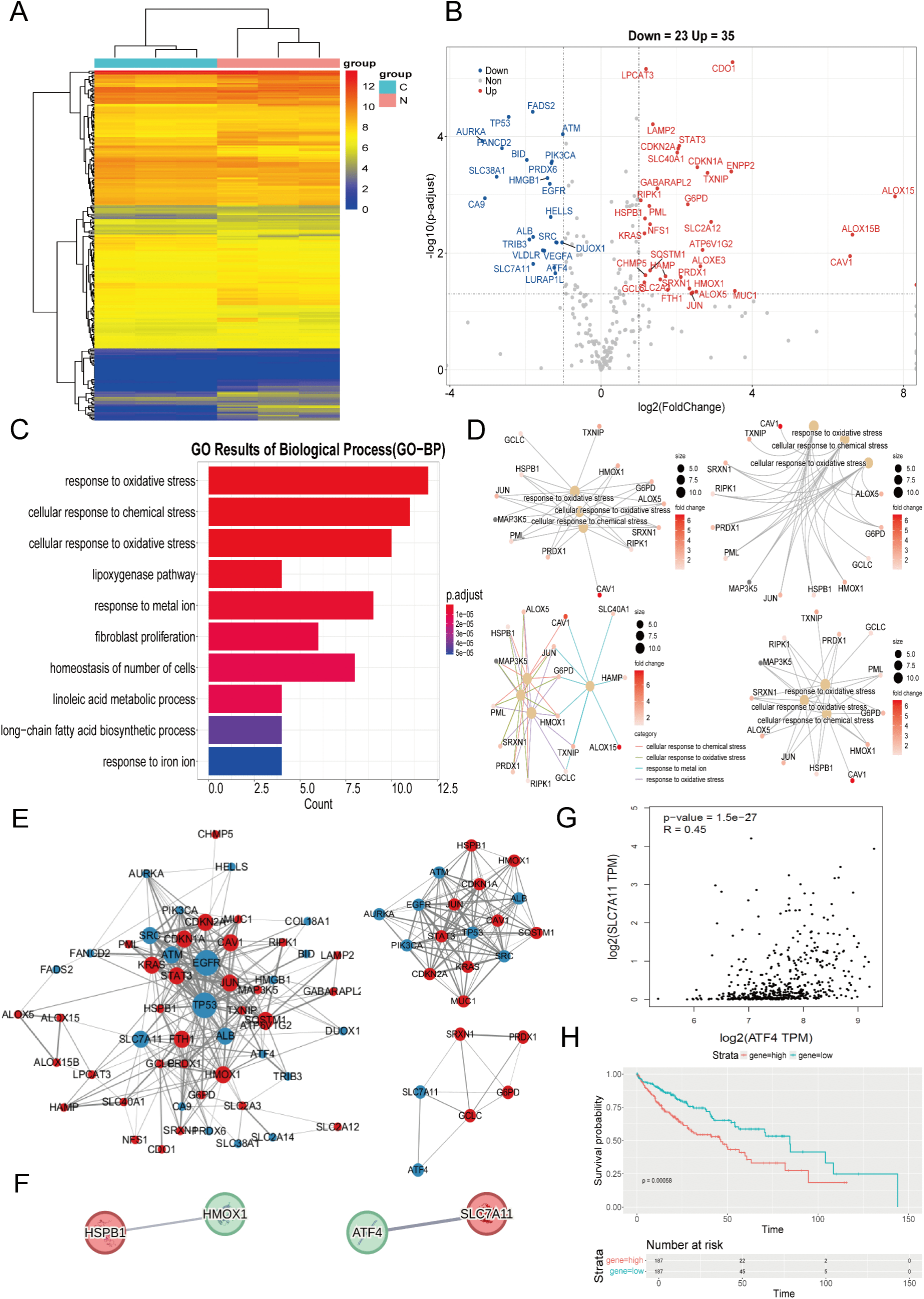
Bioinformatics screening of DEGs for ferroptosis in hepatoma cells before and after NaB treatment. (A) Heatmap showing ferroptosis-related gene expression in HepG2 cells following NaB treatment. (C: Control; N: NaB) (B) Volcano plot illustrating DEGs associated with ferroptosis in HepG2 cells after NaB treatment. (C, D) GO-BP enrichment analysis and network diagram of the enriched pathways linked to differentially expressed genes. (E) PPI network, with red and blue representing up-regulated and down-regulated genes, respectively. (F) Interaction diagrams of HSPB1/HMOX1 and ATF4/SLC7A11. (G) Scatter plot depicting the correlation between the expression levels of ATF4 and SLC7A11. (H) Kaplan-Meier survival analysis curve for ATF4 in hepatoma.

With the aim of more deeply examining the mechanism underlying NaB-induced inhibition of hepatoma cell proliferation, we initially tested ferroptosis. Ferroptosis-related genes before and after NaB treatment were identified from the GSE213944 dataset using RStudio software, and their relative expression levels were visualized through a heatmap (Fig 2A). DEGs associated with ferroptosis were displayed in a volcano plot (P-adjust < 0.05, Log2 FoldChange > 1). Among them, 35 genes showed increased expression and 23 genes showed decreased expression (Fig 2B). GO-BP enrichment analysis results (Fig 2C) indicated that NaB’s inhibitory effects on hepatoma cell proliferation may be linked to oxidative stress and intracellular iron metabolism. A network diagram of the enriched pathways and differential genes is shown in Fig 2D.

The 58 ferroptosis-related DEGs were input into the STRING database and examined with Cytoscape software to construct a PPI network. The central protein interaction network was extracted using MCODE (Fig 2E), identifying key ferroptosis marker genes that drive or inhibit ferroptosis: HSPB1/HMOX1 and ATF4/SLC7A11 (Fig 2F). Among these, ATF4/SLC7A11, with a higher interaction score (0.728) compared to HSPB1/HMOX1 (0.518), was selected for further analysis. To explore the roles and correlations of ATF4 and SLC7A11 in hepatoma, scatter plots of their expression levels were generated using the GEPIA2 website (Fig 2G), and survival analysis (P = 0.00058, < 0.001) for ATF4 was conducted using RStudio software (Fig 2H), the hazard ratio is 1.838 (S1 Table). ATF4 expression exhibited a positive relevance to the SLC7A11 expression in hepatoma, with high ATF4 expression correlating with shorter survival and poorer prognosis in patients with hepatoma.

### NaB induces ferroptosis in hepatoma cells through ATF4/SLC7A11 pathway

**Fig 3:**
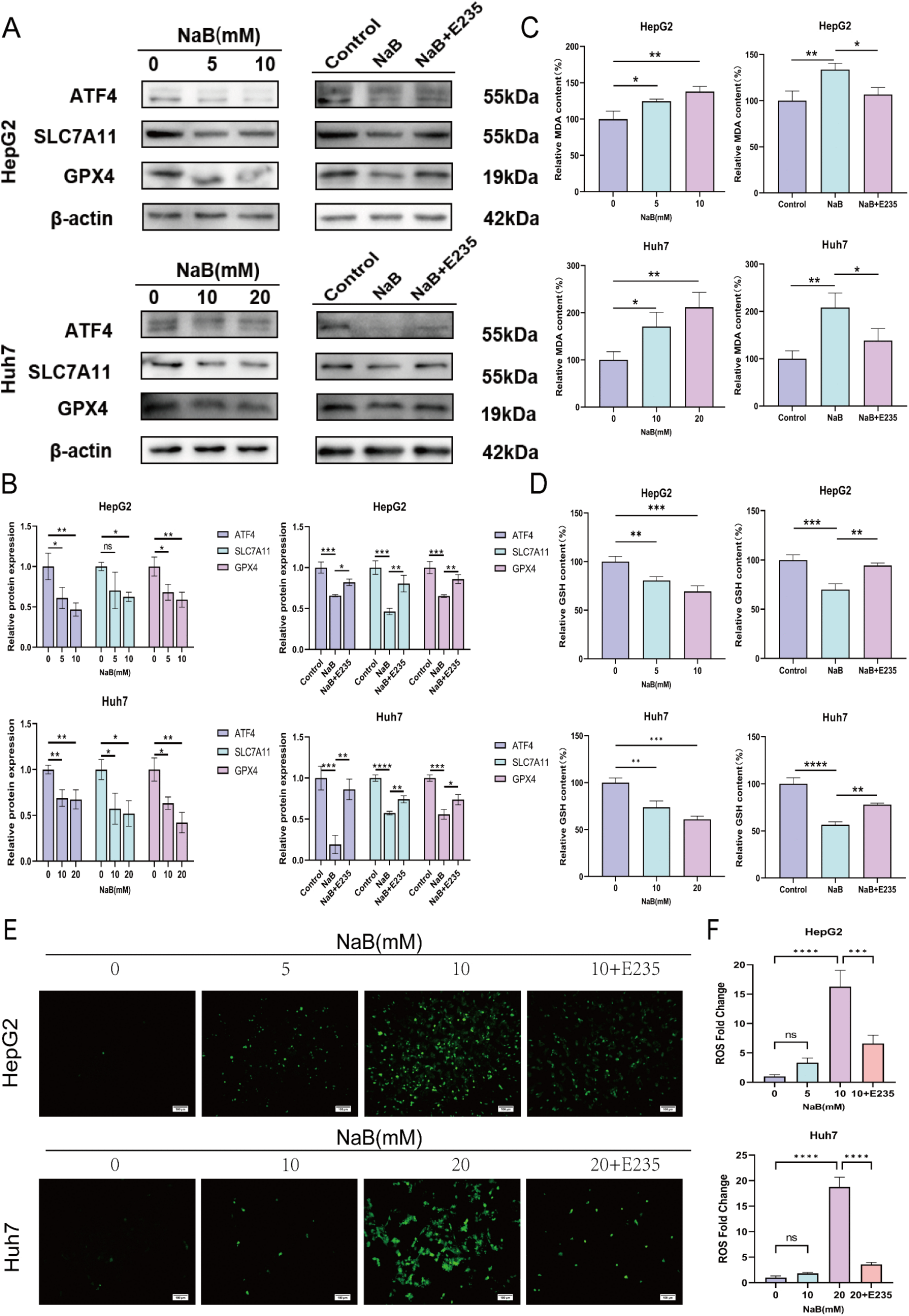
NaB induces ferroptosis in hepatoma cells throught ATF4/SLC7A11 pathway. (A,B) Western blot analysis assessing the expression levels of ATF4, SLC7A11, and GPX4 in HepG2 and Huh7 cells treated with varying NaB concentrations for 48h and in control, NaB (HepG2: 10 mM, Huh7: 20 mM), and NaB + E235 groups. (C, D) Measurement of MDA (C) and GSH (D) levels in HepG2 and Huh7 cells under different NaB concentrations for 48h, as well as in control, NaB (HepG2: 10 mM, Huh7: 20 mM), and NaB + E235 (10 μM) treatment groups. (E, F) Detection and quantification of intracellular ROS levels. Scale bar  =  100 μm. The presentation of results follows mean  ±  standard deviation (SD) format, with the number of biological replicates (n) = 3, One-way ANOVA was conducted for (Panel B-D, F).* p  <  0.05, ** p  <  0.01, *** p  <  0.001, **** p  <  0.0001.

The way NaB treatment affected the expression of ATF4 and SLC7A11 as ferroptosis-related targets was verified at the cellular level, alongside an analysis of the downstream protein GPX4. The experiment introduced the ATF4 activator E235, which enhances ATF4 expression by initiating integrated stress response (ISR) and DNA damage response signaling [21]. According to western blot analysis (Fig 3A and 3B), NaB group presented remarkably lower expression of ATF4, SLC7A11, and GPX4 in both HepG2 and Huh7 cells versus the control group. In contrast, when E235 was introduced in the NaB + E235 group, ATF4 expression increased, and the expressions of SLC7A11 and GPX4 also elevated versus the NaB-only group, further corroborating that NaB exerts its effects through the ATF4/SLC7A11 pathway.

Ferroptosis features excessively accumulated intracellular lipid peroxides and ROS. To assess these, cellular levels of glutathione (GSH) and the membrane lipid peroxide malondialdehyde (MDA) were measured using specific kits, and reactive oxygen species (ROS) levels were detected with the DCFH-DA probe. According to Fig 3C and 3D, NaB treatment led to increased MDA and decreased GSH, with both returning to baseline levels following E235 addition. Similarly, intracellular ROS content (Fig 3E and 3F) was elevated after NaB treatment, while the NaB + E235 group showed dramatically reduced ROS levels versus the NaB-only group. Taken together, NaB induces ferroptosis in HepG2 and Huh7 hepatoma cells through the ATF4/SLC7A11 pathway.

### NaB-induced ferroptosis triggers mitochondrial damage

**Fig 4:**
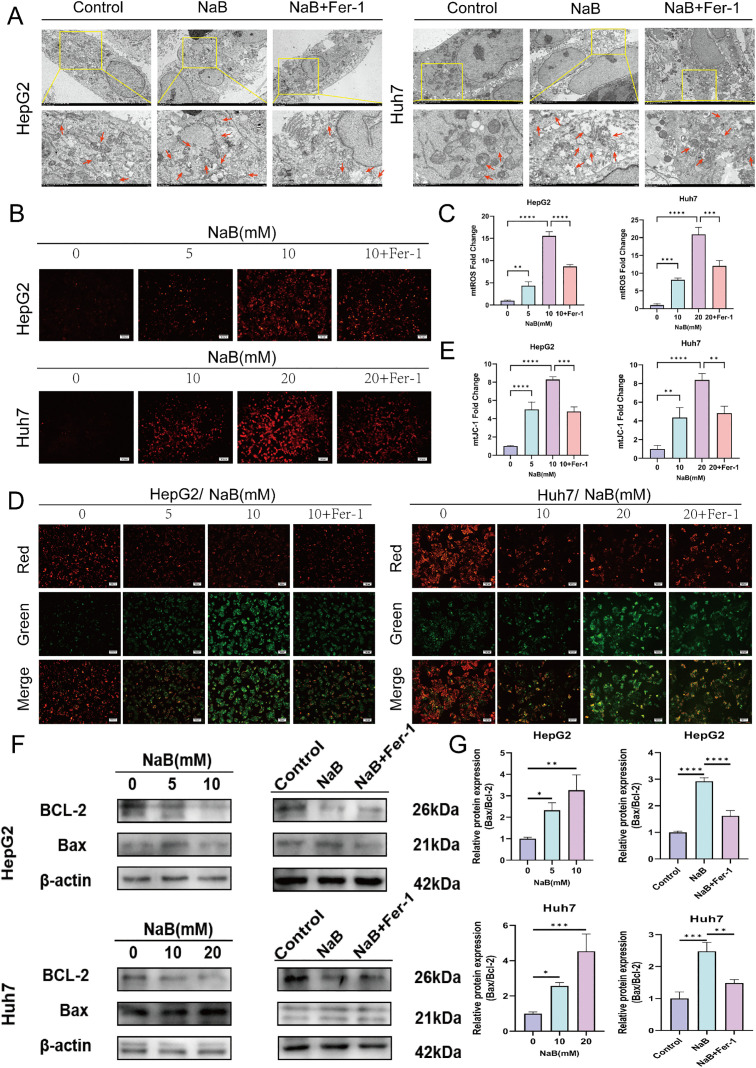
NaB-induced ferroptosis triggers mitochondrial damage. (A) Morphological changes in mitochondria after treatment with NaB for 48h at concentrations of 10 mM for HepG2 and 20 mM for Huh7. Arrows indicate damaged mitochondria. (B, C) mtROS assay results. Scale bar  =  100 μm. (D, E) MMP detection, with red and green fluorescence indicating mitochondria with normal MMP and decreased MMP respectively, Scale bar  =  100 μm; statistical analysis of these observations is presented in panel (E). (F) Expression alternation of BAX and BCL-2 following treatment with various NaB concentrations for 48h, as well as NaB (HepG2: 10 mM, Huh7: 20 mM) and NaB + Fer-1 (2 μM) groups. (G) Statistical analysis of the BAX/BCL-2 ratio. The presentation of results follows mean  ±  standard deviation (SD) format, with the number of biological replicates (n) = 3, One-way ANOVA was conducted for (Panel C,E and G).* p  <  0.05, ** p  <  0.01, *** p  <  0.001, **** p  <  0.0001.

Mitochondria, as the primary site of intracellular ROS production, are critically affected by the excessive accumulation of ROS during ferroptosis, injuring the mitochondrial oxidative respiratory chain and altering mitochondrial morphology and function. TEM results (Fig 4A) revealed that mitochondria in HepG2 and Huh7 hepatoma cells exhibited severe swelling, fractured and reduced cristae, and ruptured mitochondrial membranes with increased permeability following NaB treatment. In contrast, the extent of mitochondrial damage was attenuated in the NaB + Fer-1 group versus the NaB group. Consistent with whole-cell ROS measurements, the levels of mitochondrial ROS (mtROS) (Fig 4B and 4C) were elevated following NaB treatment and decreased in the NaB + Fer-1 group. Further analysis of MMP (Fig 4D) showed that NaB treatment decreased the number of mitochondria with normal MMP (red fluorescence) and increased those with decreased MMP (green fluorescence), with the ratio of these populations reflecting the degree of mitochondrial dysfunction. Quantitative analysis (Fig 4E) revealed a concentration-dependent increase in mitochondrial damage after NaB treatment, which was alleviated by Fer-1 administration. Western blot results (Fig 4F and 4G) demonstrated a higher BAX/BCL-2 ratio, a marker of apoptosis susceptibility, after NaB treatment, a change that could be reversed by Fer-1. Collectively, these results confirm that NaB-induced ferroptosis triggers mitochondrial damage and enhances mitochondrial membrane permeability.

### Mitochondrial damage induces mitochondrial pathway apoptosis

**Fig 5:**
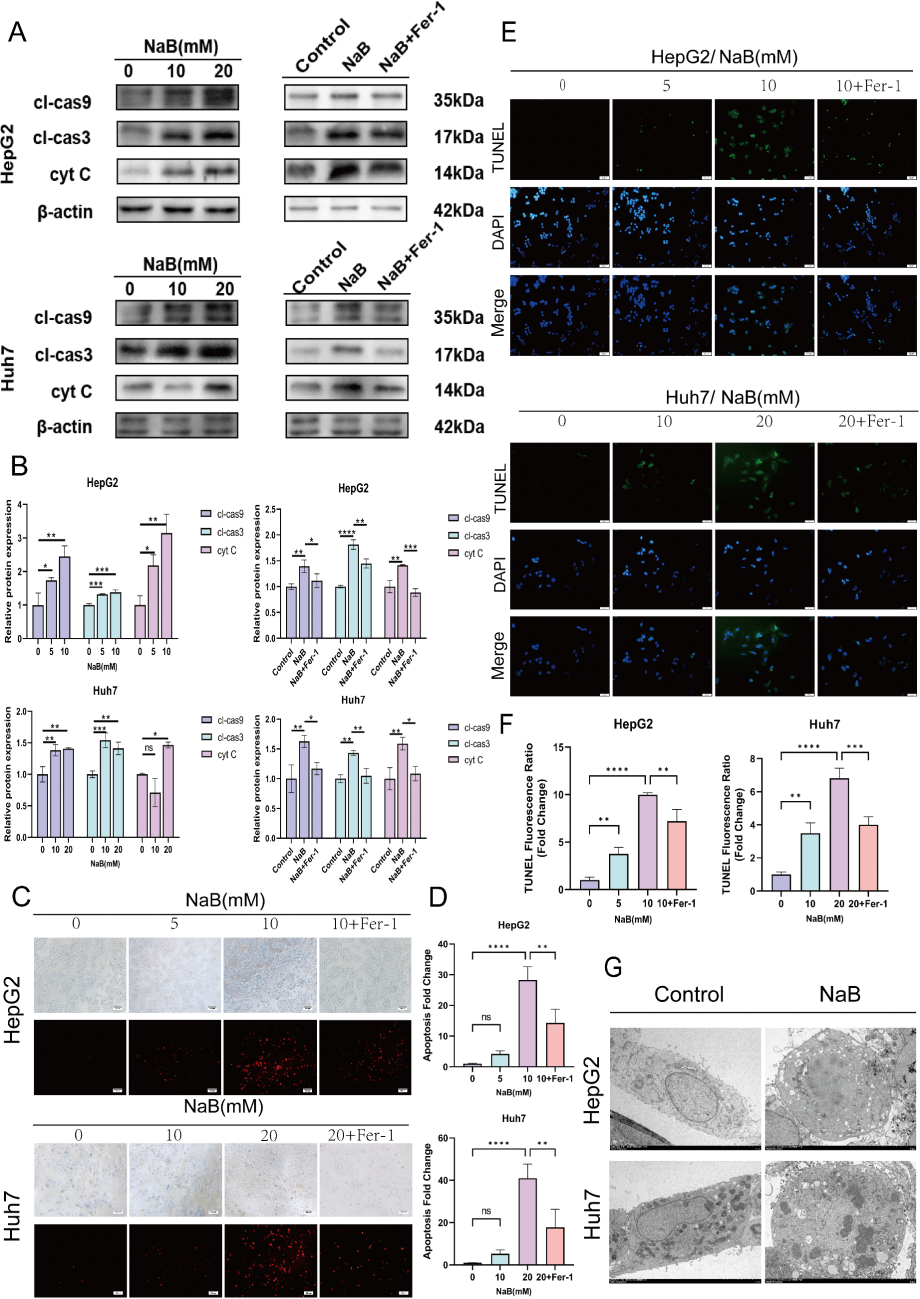
Mitochondrial damage induces mitochondrial pathway apoptosis. (A, B) Western blot analysis to assess the expressions of mitochondrial apoptotic proteins under different NaB concentrations for 48h and control, NaB (HepG2: 10 mM, Huh7: 20 mM), and NaB++ Fer-1 groups. (C, D) Annexin V-PE Apoptosis Kit used to detect PS flipping. Scale bar  =  100 μm. (E, F) TUNEL assay to identify DNA fragmentation. Scale bar  =  50 μm. (G) Morphological examination of apoptotic cells following NaB treatment (HepG2: 10 mM, Huh7: 20 mM). The presentation of results follows mean  ±  standard deviation (SD) format, with the number of biological replicates (n) = 3, One-way ANOVA was conducted for (Panel B,D and F). * p  <  0.05, ** p  <  0.01, *** p  <  0.001, **** p  <  0.0001.

Following mitochondrial damage, cytochrome C, a key component of the oxidative respiratory chain, is released into the cytoplasm through the outer mitochondrial membrane, which exhibits increased permeability and activates downstream caspase, ultimately bringing into apoptosis *via* the mitochondrial pathway. To investigate this process, the expression levels of mitochondrial apoptotic proteins (cytochrome C, cleaved caspase-9, and cleaved caspase-3), were assessed. Additionally, early-stage apoptosis was monitored by Annexin V-PE staining to detect phosphatidylserine (PS) exposure outward, and DNA fragmentation was evaluated using the TUNEL assay. Observation of apoptotic cells relied on TEM. According to the experimental results, NaB group presented obviously higher expression of mitochondrial apoptotic proteins versus the control group (Fig 5A and 5B). Moreover, the number of cells exhibiting PS externalization (Fig 5C and 5D) and DNA fragmentation detected by the TUNEL assay (Fig 5E and 5F) were both elevated following NaB treatment. However, when Fer-1 was co-incubated with NaB, all of these indicators were reduced. This observation suggests that inhibition of ferroptosis mitigates mitochondrial pathway-induced apoptosis, implying that NaB-induced apoptosis in hepatoma cells is primarily driven by mitochondrial damage resulting from ferroptosis. TEM detected rounded morphology, cytoplasmic and chromatin condensation, and nuclear rupture in apoptotic cells after NaB treatment (Fig 5G).

### NaB induces mitochondrial pathway apoptosis in hepatoma cells via ferroptosis in vivo

**Fig 6:**
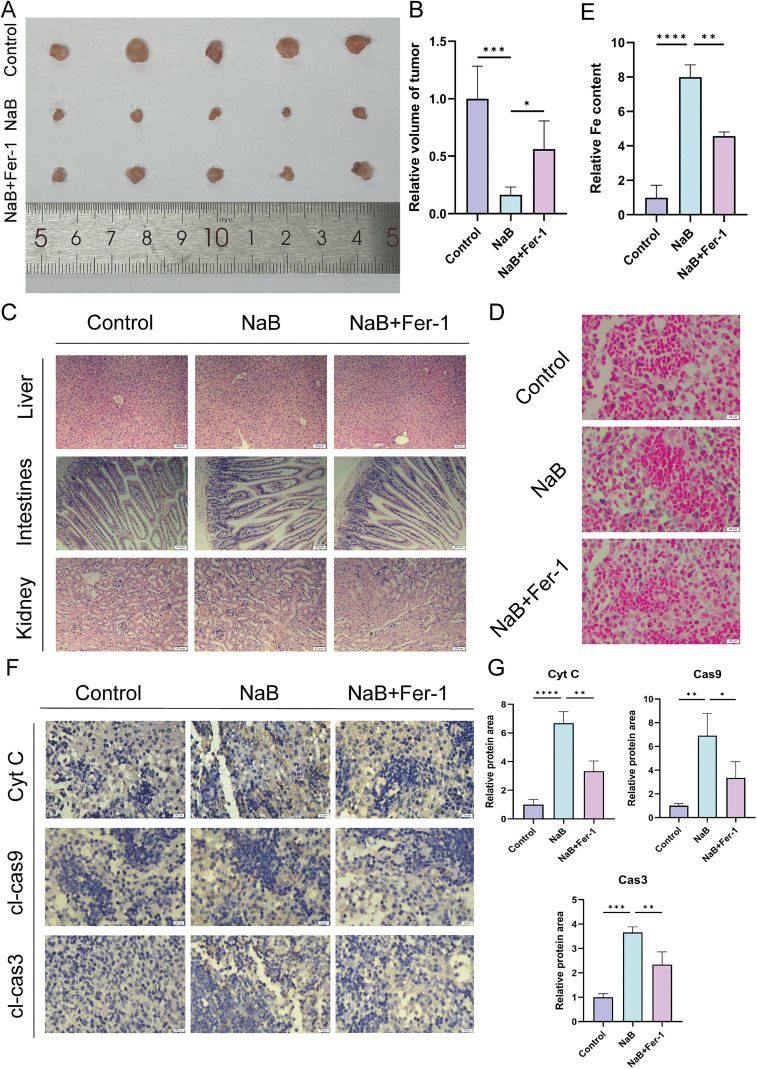
NaB induces mitochondrial pathway apoptosis in hepatoma cells *via* ferroptosis *in vivo.* (A) Tumor size assessment, n = 5. (B) Quantification of tumor volume for each group, the number of biological replicates (n) = 5. (C) HE staining to evaluate liver, kidney, and intestinal histomorphology in each group. (D, E) Prussian blue staining demonstrating changes in iron content and its quantification across the experimental groups. (F, G) IHC staining to detect the expression of mitochondrial apoptotic proteins in tumor tissues from each group. Results are presented in the form of mean ± standard deviation (SD), One-way ANOVA was conducted for (Panel B, E, and G. * p  <  0.05, ** p  <  0.01, *** p  <  0.001, **** p  <  0.0001. Scale bar  =  20 μm.

To assess whether NaB triggered mitochondrial pathway apoptosis in hepatoma cells *via* ferroptosis *in vivo*, nude mice were administered Huh7 cells by subcutaneous injection. After tumors became visible, the mice fell into the Control, NaB, and NaB + Fer-1 groups in a random manner and were treated for 14 days. The weight data of the mice during the treatment period indicated that no malnutrition occurred (S1A and S1B Figs). According to tumor size analysis results, the NaB group presented smaller tumors versus the Control group, while the NaB + Fer-1 group presented larger tumor volume versus the NaB group (Fig 6A and 6B). HE staining (Fig 6C) demonstrated that liver, kidney, and intestinal tissues in all groups maintained normal cell morphology and structure, suggesting that NaB did not induce toxicity in these organs. Prussian blue staining (Fig 6D and 6E) was employed to detect intracellular iron content, showing a marked increase in iron levels in the NaB group versus the Control group, confirming the occurrence of ferroptosis. In contrast, the total iron content in the NaB + Fer-1 group was significantly reduced, indicating inhibition of ferroptosis. In addition, IHC analysis revealed that the expression of ferroptosis protein decreased in the NaB group (S1C and S1D Figs), while the expression of mitochondrial apoptotic protein was significantly upregulated (Fig 6F and 6G). However, these decreases and increases were attenuated when ferroptosis was inhibited in the NaB + Fer-1 group. Collectively, NaB induces mitochondrial pathway apoptosis in hepatoma cells through ferroptosis *in vivo*.

## Discussion

The development of cancers, such as hepatoma, is closely linked to disruptions in internal homeostasis, and such balance can be well maintained by SCFAs from the intestinal microbiota [7,22]. Consequently, research into the role of SCFAs in cancer has expanded significantly, with a particular focus on colorectal cancer. In this context, cancer inhibition mechanisms may rely on the suppression of inflammation and the enhancement of the intestinal barrier [23–25]. SCFAs not utilized by colorectal cells are transported through apical and basolateral membranes [26,27], ultimately reaching other tissue cells *via* the bloodstream. Due to the Warburg effect, short-chain fatty acid butyrate does not act as a metabolic substrate in tumor cells. Instead, after accumulating within the cells, it acts as an HDAC inhibitor, altering the fate of cancer cells by influencing the Wnt, Hedgehog, Hippo and Notch pathways [7,28,29].

Emerging evidence suggests that short-chain fatty acids (SCFAs), including NaB, can reverse chemoresistance by restoring cancer cell susceptibility to ferroptosis [30]. While most studies on NaB in HCC have focused on its combinatorial effects with conventional chemotherapeutics—particularly its synergistic enhancement of sorafenib efficacy [31,32], the mechanistic basis of NaB monotherapy remains poorly characterized. A seminal study demonstrated that NaB monotherapy induces ferroptosis in HCC cells through GPX4 ubiquitination-mediated degradation. However, upstream regulatory mechanisms remain unexplored [33]. Our work not only confirms NaB’s ability to induce ferroptosis in HCC cells, but also establishes that NaB inhibits hepatocellular carcinoma cell proliferation by inducing both ferroptosis and apoptosis. Furthermore, we demonstrate that NaB triggers ferroptosis through ATF4/SLC7A11 axis regulation and elucidate the role of mitochondria in NaB-induced ferroptosis and apoptosis in hepatoma cells.

Ferroptosis, an iron-dependent form of cell death, differs from apoptosis. The system x_c_^-^, a critical suppressor [16], encompasses SLC7A11/xCT and SLC3A2/4F2hc/CD98hc, and primarily mediates the antiport of cystine and glutamate [34]. When system xc- is inhibited, cystine intake, which essentially participates in synthesizing the antioxidant GSH, is diminished. This significantly impairs the cell’s capability of combating oxidative stress, rendering it more susceptible to lipid peroxide and ROS-induced damage, leading to ferroptosis. According to existing studies, ATF4 modulates the transcription of SLC7A11 [20,35,36], SLC7A11 and GPX4 can be directly modulated by small molecules [37], which aligns with our findings. Accordingly, the expression of ATF4, SLC7A11, and its downstream protein GPX4 in hepatoma cells was verified following NaB treatment. The observed reduction in protein expression levels relative to the control group suggests that NaB induces ferroptosis by modulating the ATF4/SLC7A11 axis. This was further corroborated by the detection of GSH, MDA, and intracellular ROS. As an HDACi [28], NaB’s regulatory effect on ATF4 may be related to its indirect inhibition of ATF4 expression. Notably, when E235 was added, the experimental indicators presented an obvious change in statistical level compared to the NaB-only group, providing a robust validation of our findings. Previous reports have established a link between elevated ATF4 expression and the survival and progression of various cancers [38], and survival analysis data from a public hepatoma database revealed that high ATF4 expression correlates with poor prognosis. Based on these results, NaB may offer a novel therapeutic option for hepatoma treatment.

Mitochondria, as essential organelles [39], have long been studied for their involvement in ferroptosis. Research has demonstrated that mitochondria participate in the ferroptosis process, particularly in the context of cysteine depletion (converted from cystine imported *via* system x_c_^-^) the subsequent production of lipid ROS, a mechanism that aligns with the ferroptosis pathway induced by NaB in our study [40]. It is well-established that a reduction in MMP marks an early event in apoptosis initiation [41]. Furthermore, as ROS-generating organelles, mitochondria are intricately connected to the onset of apoptosis [42]. The present study, observed a decrease in MMP alongside an excessive accumulation of mtROS after NaB treatment, suggesting that NaB may induce apoptosis in hepatoma cells concurrently with ferroptosis.

The BCL-2 family of proteins typically maintains the balance engaging in preventing cells from undergoing apoptosis [43,44]. This balance can be disrupted when cells experience oxidative stress [45]. Reduced BCL-2 protein levels, with or without direct activation of BAX, triggers mitochondrial pathway apoptosis [46], and the ratio of BAX to BCL-2 can indicate the apoptosis sensitivity. This process progresses more deeply accompanied by increased MMP, which facilitates cytochrome C to be released from the oxidative respiratory chain into the cytoplasm to be bound to apoptotic peptidase activating factor 1 (APAF1) to activate precursor caspase-9, as a result, downstream effector protein caspase-3 undergoes proteolytic activation [47,48]. Other hallmarks of apoptosis (PS flip and DNA fragmentation) were examined. These findings collectively confirm that mitochondrial pathway apoptosis can be induced in hepatoma cells following NaB treatment.

**Fig 7:**
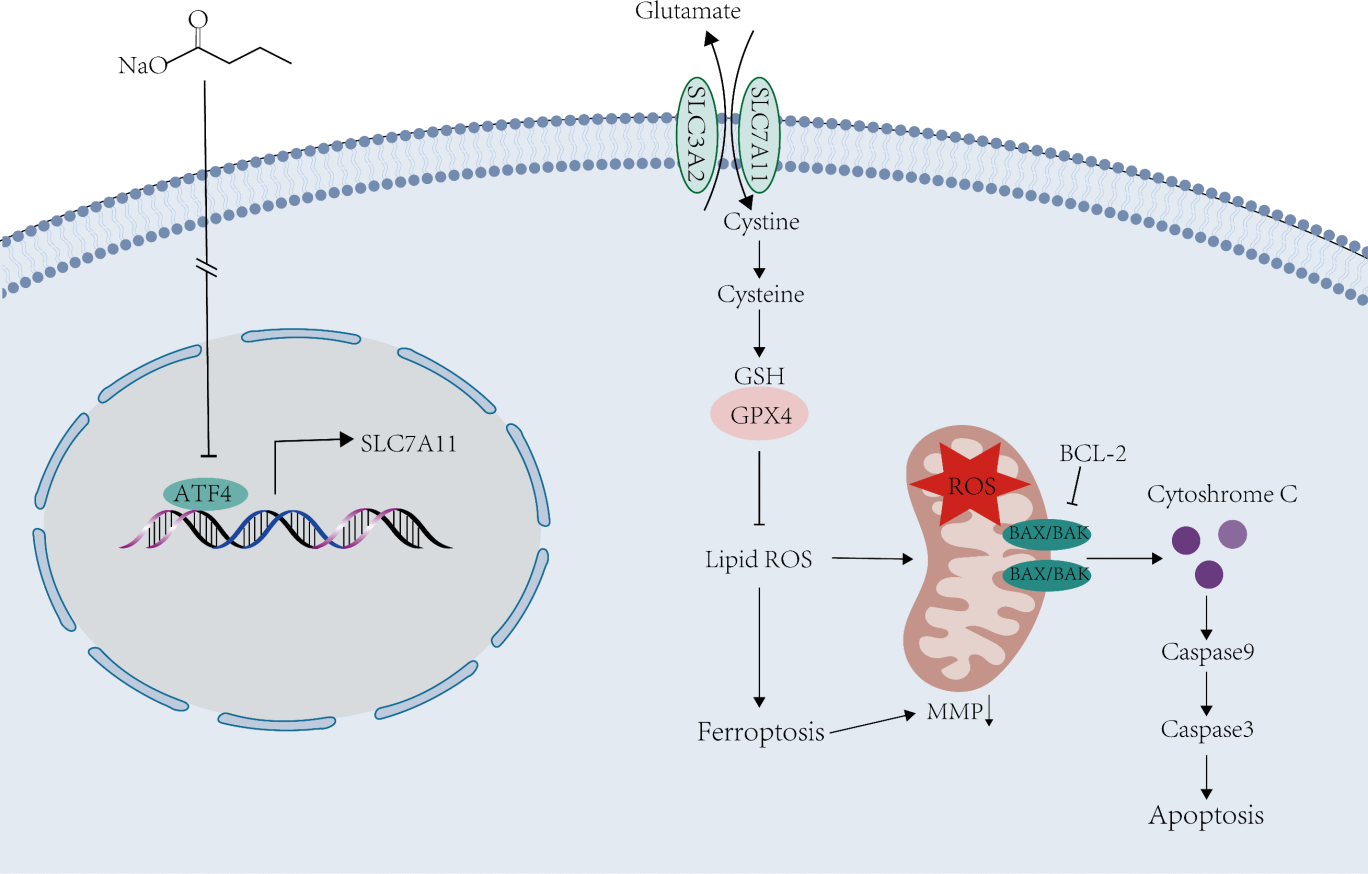
Proposed mechanism by which NaB inhibits hepatoma cell proliferation through inducing apoptosis and ferroptosis.

Taken together, NaB inhibits hepatoma cell proliferation by inducing both ferroptosis and apoptosis. This dual mechanism is initiated by ATF4/SLC7A11-induced ferroptosis, with the resulting ROS accumulation disrupting the balance between BAX and BCL-2. ROS-induced oxidative stress and the loss of MMP collectively lead to the opening of mitochondrial outer membrane pores, facilitating cytochrome C to be released into the cytoplasm, and accordingly triggering certain enzymatic reactions in mitochondrial pathway apoptosis (Fig 7). However, it remains unclear whether there is a common upstream regulator that coordinates both apoptosis and ferroptosis in response to NaB. Future research is suggested to pay special attention to investigating this potential regulatory mechanism.

## Conclusion

This study provides *in vitro* and *in vivo* evidence that NaB induces mitochondrial pathway apoptosis in hepatoma cells through the mechanism of ferroptosis, leading to the inhibition of hepatoma cell proliferation. Furthermore, the link between ferroptosis and mitochondrial pathway apoptosis is mediated by mitochondrial dysfunction. Considering the dual properties of SCFAs—both inhibiting tumor growth and protecting surrounding tissues—our findings suggest that NaB could offer promising directions for the development of safe and effective hepatoma therapies. This is consistent with the growing recognition of ferroptosis as an actionable therapeutic target in oncology [49], and our work provides mechanistic insights that may inform future strategies targeting the ferroptosis–mitochondrial apoptosis axis in liver cancer.

## Supporting information

S1 Fig NaB induces mitochondrial pathway apoptosis in hepatoma cells via ferroptosis in vivo. (A) The weight changes of mice during the treatment period, the number of biological replicates (n) = 5. (B) The body weight of mice in each group after two weeks of treatment. (C, D) IHC staining to detect the expression of ferroptosis proteins in tumor tissues from each group. Results are presented in the form of mean ± standard deviation (SD), One-way ANOVA was conducted for panels B and D.). * p  <  0.05, ** p  <  0.01, *** p  <  0.001, **** p  <  0.0001. Scale bar  =  20 μm.
(TIF)

S1 Table ATF4 expression quantity and other factors adjusted composite outcome. (DOCX)

S1 File Raw images. (PDF)

S2 File Raw material. (XLSX)
