# Supplementary material for: Sodium butyrate induces mitochondrial pathway apoptosis in liver cancer via ATF4/SLC7A11-mediated ferroptosis
Source: PLoS One. 2026 Jul 15;21(7):e0353653. doi: 10.1371/journal.pone.0353653 (PMC13372185; doi:10.1371/journal.pone.0353653)
Supplement: S1 Table — (DOCX) [file pone.0353653.s002.docx]

Table S1. ATF4 expression quantity and other factors adjusted composite outcome

| **Variable** | **Univariate analysis** | |
| --- | --- | --- |
|  | **HR(95% CI)** | ***P*-value** |
| Expression level of ATF4 | 1.838(1.29-2.613) | < 0.001 |
| gender | 1.121(0.939-1.338) | 0.207 |
| age | 1.087(0.915-1.292) | 0.344 |
| race | 1.034(0.816-1.331) | 0.783 |
