# Supplementary material for: Sodium butyrate induces mitochondrial pathway apoptosis in liver cancer via ATF4/SLC7A11-mediated ferroptosis
Source: PLoS One. 2026 Jul 15;21(7):e0353653. doi: 10.1371/journal.pone.0353653 (PMC13372185; doi:10.1371/journal.pone.0353653)
Supplement: S1 File — (PDF) [file pone.0353653.s003.pdf]

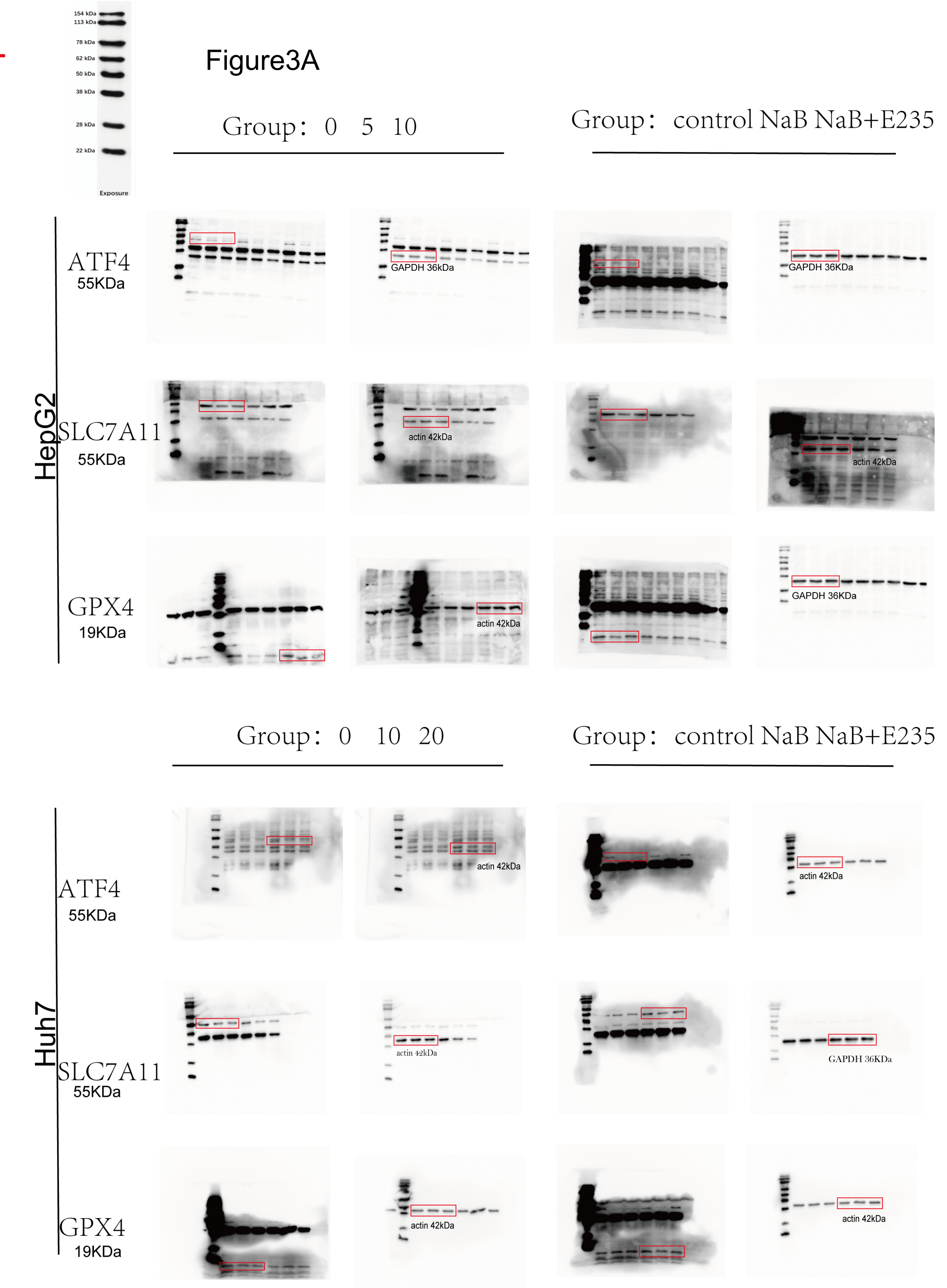
154 kDa
113 kDa
78 kDa
62 kDa
50 kDa
38 kDa
28 kDa
22 kDa
Exposure
Figure3A
Group： 0 5 10
Group： control NaB NaB+E235
HepG2
ATF4
55KDa
GAPDH 36kDa
GAPDH 36KDa
SLC7A11
55KDa
actin 42kDa
actin 42kDa
GPX4
19KDa
actin 42kDa
GAPDH 36KDa
Group： 0 10 20
Group： control NaB NaB+E235
Huh7
ATF4
55KDa
actin 42kDa
actin 42kDa
SLC7A11
55KDa
actin 42kDa
GAPDH 36KDa
GPX4
19KDa
actin 42kDa
actin 42kDa

Figure4F

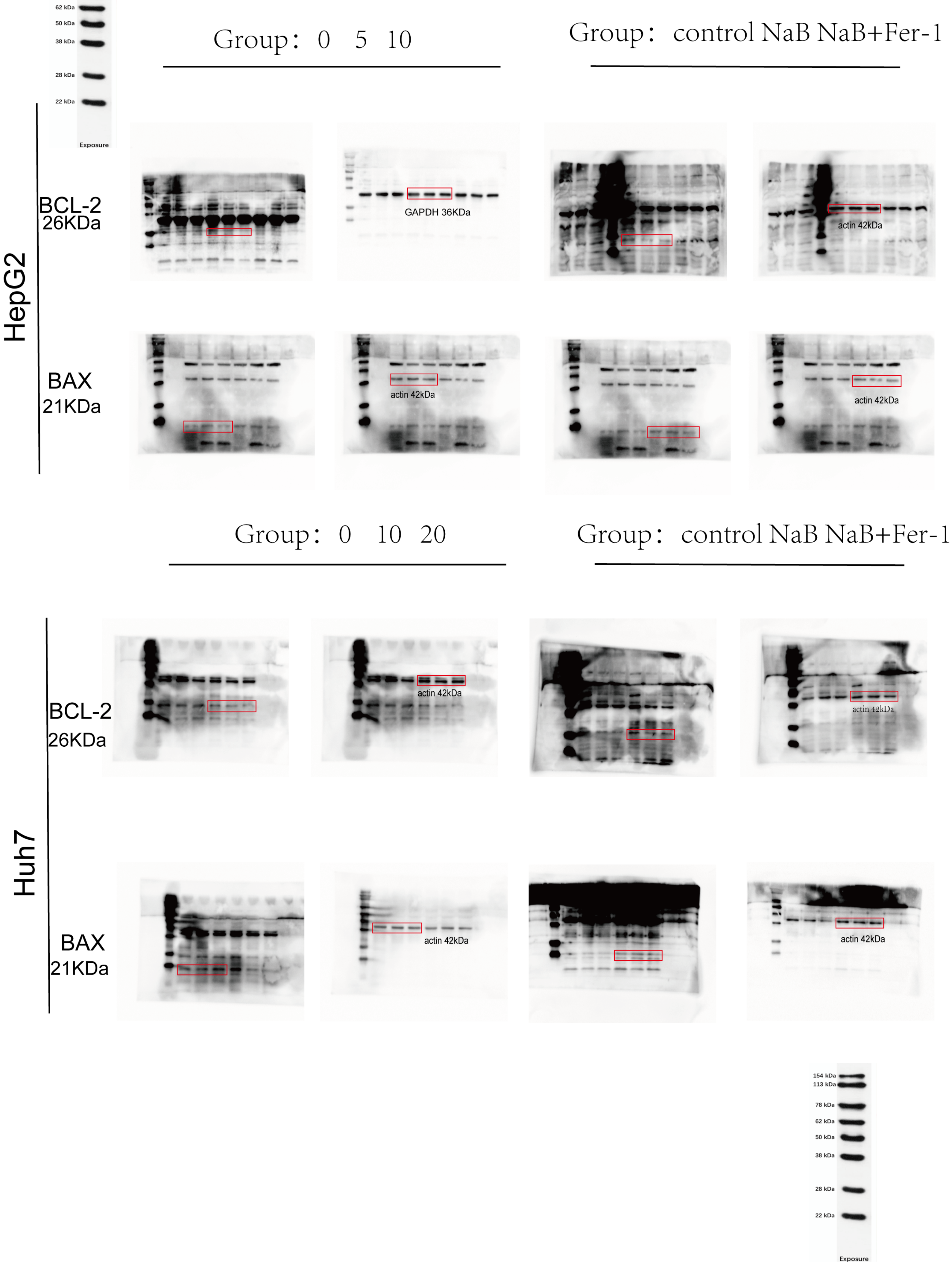
154 kDa
113 kDa
78 kDa
62 kDa
50 kDa
38 kDa
28 kDa
22 kDa
Exposure
Group：0 5 10
Group：control NaB NaB+Fer-1
HepG2
BCL-2
26KDa
GAPDH 36KDa
actin 42kDa
actin 42kDa
BAX
21KDa
actin 42kDa
actin 42kDa
Group：0 10 20
Group：control NaB NaB+Fer-1
Huh7
BCL-2
26KDa
actin 42kDa
actin 42kDa
BAX
21KDa
actin 42kDa
actin 42kDa
154 kDa
113 kDa
78 kDa
62 kDa
50 kDa
38 kDa
28 kDa
22 kDa
Exposure

Figure5A

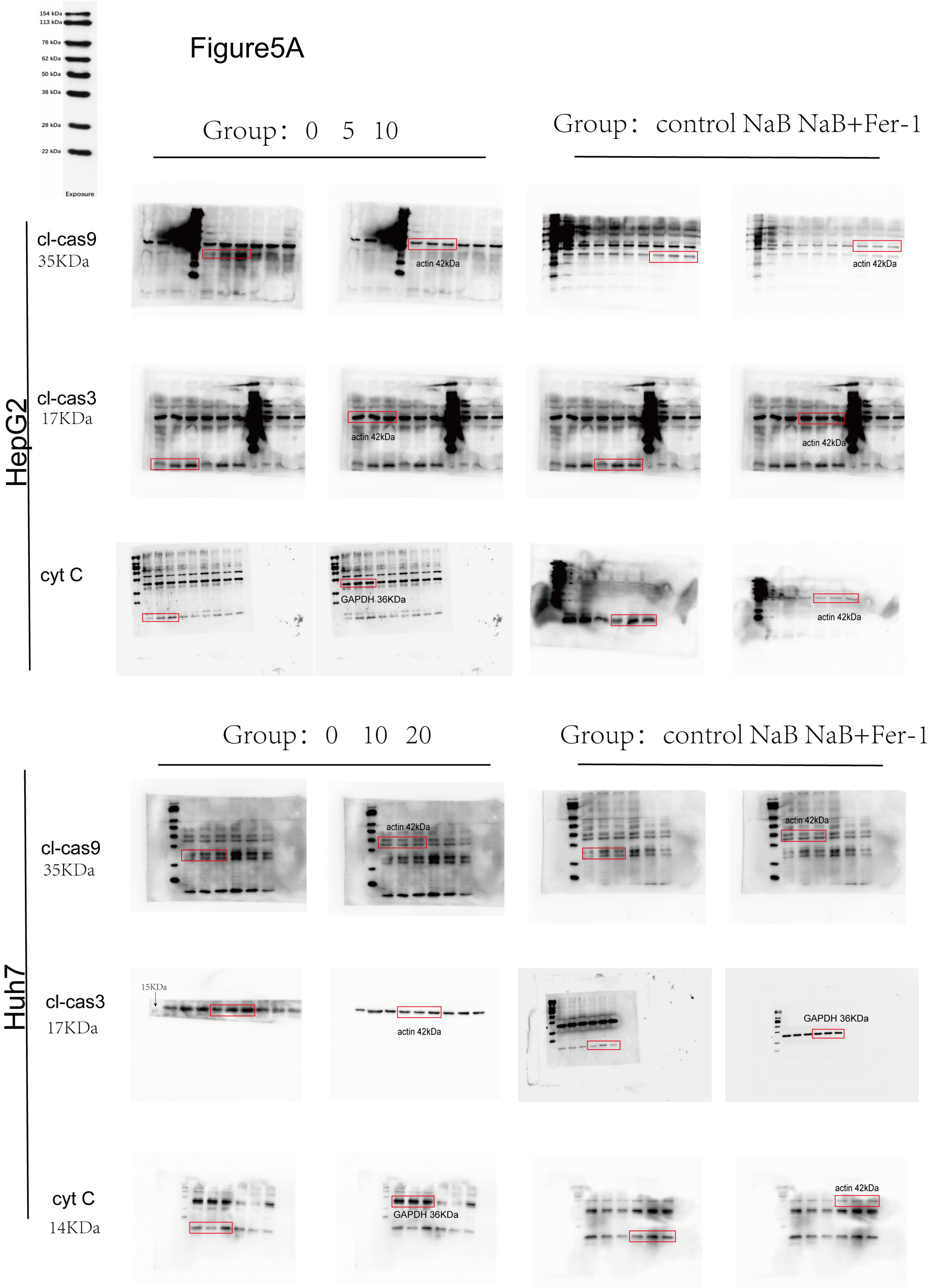
154 kDa
113 kDa
78 kDa
62 kDa
50 kDa
38 kDa
28 kDa
22 kDa
Exposure
Group： 0 5 10
Group： control NaB NaB+Fer-1
HepG2
cl-cas9
35KDa
actin 42kDa
actin 42kDa
cl-cas3
17KDa
actin 42kDa
actin 42kDa
cyt C
GAPDH 36KDa
actin 42kDa
Group： 0 10 20
Group： control NaB NaB+Fer-1
Huh7
cl-cas9
35KDa
actin 42kDa
actin 42kDa
cl-cas3
17KDa
15KDa
actin 42kDa
GAPDH 36KDa
cyt C
14KDa
GAPDH 36KDa
actin 42kDa
